# Modifiable risk factors for cancer

**DOI:** 10.1038/sj.bjc.6601509

**Published:** 2004-01-20

**Authors:** C J Stein, G A Colditz

**Affiliations:** 1 Harvard Center for Cancer Prevention, 181 Longwood Avenue, Boston, MA 02115, USA; 2 Cancer Epidemiology Program Dana-Farber/Harvard Cancer Center, 181 Longwood Avenue, Boston, MA 02115, USA; 3 Channing Laboratory, Department of Medicine, Brigham and Women's Hospital and Harvard Medical School, 181 Longwood Avenue, Boston, MA 02115, USA

**Keywords:** prevention, tobacco, physical activity, obesity, diet, screening

## Abstract

Over 6 million people around the world die from cancer each year. Modifiable risk factors have been linked to a wide range of malignancies, including cancers of the oropharynx, oesophagus, larynx, lung, kidney, bladder, pancreas, skin, stomach, ovary, breast, cervix, uterus, prostate, and colon. Research indicates that over half of all cancers in developed countries could be prevented if we implemented population-wide measures to promote the following behaviours: reduce tobacco use, increase physical activity, control weight, improve diet, limit alcohol, utilise safer sex practices, get routine cancer screening tests, and avoid excess sun exposure.

Over 6 million people around the world die from cancer each year (Murray and Lopez, 1996). There is overwhelming evidence that lifestyle factors impact cancer risk and that positive, population-wide changes can significantly reduce the cancer burden (Curry *et al*, 2003). Current epidemiologic evidence links behavioural factors to a variety of malignancies, including the most common cancers diagnosed in the developed world – lung, colorectal, prostate, and breast cancer (Ezzati *et al*, 2002). Owing to the tremendous impact of modifiable factors on risk, especially for the most prevalent cancers, it has been estimated that 50% of cancer is preventable (Colditz *et al*, 1996). However, to bring about dramatic reductions in cancer incidence, widespread lifestyle changes are necessary.

Multiple observations attest to the success and marked benefit of population-wide prevention strategies. For example, the reductions in lung cancer rates in the US, seen first in young men, then in older men, and finally in women, mirror changes in cigarette smoking patterns (Wingo *et al*, 1999). Introduction of the Papanicolaou test in the 1950s was followed by a dramatic decline in cervical cancer in those countries that made widespread screening available (Laara *et al*, 1987). The decline in Australian melanoma mortality for those born after 1950 is an additional example of effective intervention at the population level (Giles *et al*, 1996).

Behaviour change is possible and offers great potential for cancer prevention. This paper summarises the major factors that can be modified to decrease cancer risk. Current recommendations include reducing tobacco use, increasing physical activity, controlling weight, improving diet, limiting alcohol, utilising safer sex practices, getting routine cancer screening tests, and avoiding excess sun exposure. Many of these cancer prevention strategies not only reduce the risk of multiple cancers but also significantly influence the risk of other chronic diseases. Global mortality data are included as an indication of the general impact of each lifestyle factor on health.

## TOBACCO PREVENTION AND CESSATION

Tobacco is a major cause of preventable death around the world, accounting for nearly 5 million deaths each year (Ezzati *et al*, 2002). Approximately half of all smokers die of tobacco-related disease(Doll *et al*, 1994), and in the US, adult smokers lose an average of over 13 years of life because of the negative consequences of smoking (Centers for Disease Control and Prevention, 2002).

Smoking contributes to approximately 30% of all cancers in the developed world, causing over 90% of lung cancers in addition to a large range of other malignancies, such as cancers of the mouth, larynx, oesophagus, pancreas, stomach, colon, cervix, kidney, and bladder. Growing evidence also ties smoking to an elevated risk of liver and prostate cancer, as well as leukaemia. Tobacco likely acts on multiple stages of carcinogenesis; it not only delivers a host of carcinogens but also causes irritation and inflammation and interferes with the body's natural protective barriers.

The health risks of tobacco use are not limited to cigarette smoking. Cigar, pipe, and smokeless tobacco use also increase the risk of cancer, as does exposure to environmental tobacco smoke (secondhand smoke). In addition to malignancy, smoking causes many other diseases and conditions, including heart disease, stroke, lung infection, and pregnancy complications (Ezzati *et al*, 2002). However, many of the risks associated with tobacco use fall rapidly after cessation.

To decrease the global burden of cancer and other chronic diseases it is essential to implement programs and policies that reduce youth initiation and facilitate smoking cessation in clinical and community settings (Curry *et al*, 2003).

## PHYSICAL ACTIVITY

The population in the US and many other developed countries is remarkably inactive: over 60% of the US adult population does not participate in regular physical activity, and this estimate includes 25% of people who are almost entirely sedentary (US Department of Health and Human Services *et al*, 1999). Globally, inactivity causes close to 2 million deaths each year (Ezzati *et al*, 2002). It is linked to most major chronic diseases, including type II diabetes, osteoporosis, stroke, cardiovascular disease, and cancer.

Inactivity increases the risk of colon and breast cancer and likely endometrial cancer as well (International Agency for Research on Cancer, 2002). The impact on colon cancer risk is especially striking; high levels of physical activity may reduce the risk of colon cancer by as much as 50% (Colditz *et al*, 1997; International Agency for Research on Cancer, 2002). Growing evidence suggests that lack of physical activity also may be associated with an elevated risk of lung and prostate cancer. Overall, sedentary lifestyle has been linked to 5% of deaths from cancer (Colditz *et al*, 1996).

The associations between physical activity and cancers of the colon and breast have been documented across levels of obesity, suggesting that physical activity acts on cancer risk independent of its effects on body weight. Several mechanisms have been proposed to explain the dose–response relationship observed between activity and cancer risk. First, physical activity may reduce circulating levels of insulin, hormones, and other growth factors (McKeown-Eyssen, 1994; Giovannucci *et al*, 1995). Physical activity may also alter prostaglandin levels and improve immune function (Martinez *et al*, 1999). In the case of colon cancer, modification of bile acid metabolism may lower risk (Martinez *et al*, 1999), and it has been hypothesised that by decreasing gastrointestinal transit time, physical activity can also minimise contact time between the colonic mucosa and potential carcinogens in the stool (McTiernan *et al*, 1998).

Fortunately, the negative effects of a sedentary lifestyle are reversible: evidence shows that, even after years of inactivity, increased physical activity can reduce the risk of premature death (Paffenbarger *et al*, 1993). As little as 30 min a day of moderate physical activity (such as brisk walking) significantly reduces disease risk (Pate *et al*, 1995).

Beyond individual behaviour choice, changes are needed at the family, community, and organisational levels to create an environment that is safe for and conducive to physical activity.

## WEIGHT CONTROL AND OBESITY PREVENTION

As a result of pervasive changes in diet and physical activity patterns, obesity is increasing at epidemic rates around the world (International Agency for Research on Cancer, 2002) and is estimated to account for over 2.5 million deaths each year (Ezzati *et al*, 2002). Currently, almost 65% of American adults are overweight, and over 30% are considered obese (Flegal *et al*, 2002). Although certain segments of the population have experienced a higher rate of increase than others, this epidemic has affected people of all ages, races, ethnicities, socioeconomic levels, and geographic locations.

Excess weight alters levels of hormones and growth factors. It also causes severe health consequences. Overweight and obesity cause many types of cancer, including colorectal, postmenopausal breast, endometrial, renal, and oesophageal cancer, with a population attributable risk that ranges from 9% (postmenopausal breast cancer) to 39% (endometrial cancer) (International Agency for Research on Cancer, 2002). In addition to these cancers, a recent study by Calle *et al* (2003) suggests that obesity also may influence cancers of the prostate, liver, gallbladder, pancreas, stomach, ovary, and cervix in addition to non-Hodgkin's lymphoma and multiple myeloma. Overall, obesity may cause 14% of cancer deaths in men and 20% of cancer deaths in women.

In addition to this large impact on cancer, overweight and obesity also increase the risk of a multitude of other diseases and conditions, such as stroke, cardiovascular disease, type II diabetes, osteoarthritis, and pregnancy complications.

The strategy behind most weight control measures is to create a balance between caloric intake through diet and energy expenditure through physical activity. The International Agency for Research on Cancer (IARC) has proposed a comprehensive set of recommendations for public health action on weight control. These guidelines recommend intervention at multiple levels, including health care provider involvement, regulation to ensure access to safe places for physical activity (including school, worksite, and community), and family and community interventions (International Agency for Research on Cancer, 2002).

## DIETARY IMPROVEMENTS

A great deal of research has focused on identification of a diet-cancer connection, and many different factors have been investigated.

In terms of cancer and chronic disease prevention, a healthy diet is one that is rich in fruits and vegetables, is limited in red meat and animal fat, and includes a daily multivitamin with folate (Colditz *et al*, 1996).

### Fruits and vegetables

Fruits and vegetables contain a variety of healthy components, including vitamins, minerals, and fibre. Consumption of fruits and vegetables has been shown to decrease the risk of cardiovascular disease and type II diabetes (World Cancer Research Fund and American Institute for Cancer Research, 1997), and the global burden of inadequate intake is estimated to account for more than 2.7 million deaths each year (Ezzati *et al*, 2002). Although early evidence suggested a strong link between fruits, vegetables, and cancer risk, recent data from prospective cohort studies suggest only weak associations (Feskanich *et al*, 2000; Smith-Warner *et al*, 2001). Total intake may affect the risk of cancers of the pancreas, bladder, lung, colon, mouth, pharynx, larynx, oesophagus, and stomach (Curry *et al*, 2003).

In addition to total intake, researchers have also examined the effects of specific fruits and vegetables on cancer risk, and the relationship between prostate cancer and tomatoes appears to be the most promising (Giovannucci, 1999). Four cohort studies have reported on this association, and each of them demonstrated a 40–50% prostate cancer risk reduction among men who consumed the highest amounts of tomatoes and tomato products. It is postulated that the carotenoid lycopene may be responsible for this protective effect.

### Folate

Folate, a B vitamin, is important in the synthesis, methylation, and repair of DNA, and a number of studies have found that as folate intake increases, the risk of adenomatous polyps and colorectal cancer decreases (Freudenheim *et al*, 1991; Giovannucci *et al*, 1993). Folate's interactions with different forms of methylenetetrahydropholate reductase add additional support to this causal relationship (Slattery *et al*, 1999). Growing evidence also suggests that folate reduces the adverse effects of alcohol on breast cancer risk (Zhang *et al*, 2003).

The Nurses' Health Study found that a high intake of folate from fruits and vegetables lowers colorectal cancer risk, but supplementation with a multivitamin containing folate offers even greater risk reduction (Giovannucci *et al*, 1998). Based on these findings and the clear benefits for prevention of neural tube defects and cardiovascular disease, use of a daily vitamin supplement containing folate is recommended.

### Vitamin A and carotenoids

Important in cell division and cell differentiation, vitamin A and carotenoids have been studied extensively for their potential impact on cancer risk. Existing evidence indicates that there is a small reduction in the risk of breast cancer associated with a high intake of carotenoids (Zhang *et al*, 1999). Although a number of observational studies have suggested an association between carotenoid intake and a decreased risk of lung cancer, randomised trials of beta-carotene intake found either no effect or an elevated risk of lung cancer (Hennekens *et al*, 1996; Omenn *et al*, 1996).

### Selenium

Animal studies suggest that higher intakes of selenium reduce the risk of various tumours, and ecologic studies show an inverse relation between selenium and cancers of the breast and colon (Clark, 1985). A randomised trial of selenium to reduce skin cancer risk showed a significant reduction in cancers of the lung, colon, and prostate (Clark *et al*, 1996). Selenium may offer this protection because it is a key part of antioxidant enzymes and an important element in immune system function. However, evidence against a large and rapid impact of selenium on cancer risk comes from the fortification intervention that was implemented in Finland. Owing to the low selenium levels in the soil (leading to low selenium levels in foods), selenium was applied with fertilizer in the mid-1980s. Although blood selenium levels rose rapidly following this ecologic intervention, there has been no apparent decline in incidence or mortality rates for prostate or colon cancer (Willett, 1999).

### Fats

Despite previous hypotheses, total fat intake does not seem to alter cancer risk. However, all fats are not the same, and there are some data to suggest that diets high in animal fat raise the risk of prostate and colorectal cancer. Different types of fat are known to impact the risk of cardiovascular disease; saturated fats and trans fatty acids raise risk, while unsaturated fats lower it.

### Red meat

High intake of red meat, including beef, pork, lamb, and veal, is associated with an increased risk of colorectal cancer. The mechanism of this elevated risk is unclear, but may be related to the high concentration of animal fat or may be associated with carcinogens produced during the cooking of animal proteins at high temperatures.

### Fibre

Long believed to play a role in colorectal cancer prevention, fibre does not appear to have a significant impact on cancer risk. It does, however, decrease the risk of other chronic diseases such as cardiovascular disease and type II diabetes.

## LIMITATION OF ALCOHOL USE

Excess alcohol intake is responsible for more than 1.8 million deaths each year (Ezzati *et al*, 2002). Data have emerged on the benefits of moderate alcohol use in terms of reducing cardiac and diabetes risk, but alcohol remains a risk factor for cancer mortality (Thun *et al*, 1997). Alcohol use is a primary cause of oesophageal and oral cancer, and even moderate intake is associated with an increased risk of breast and colorectal cancer. Persistent, heavy alcohol use has been linked to elevated liver cancer risk. In addition to contributing to a higher risk of malignancy, there are a variety of other health risks associated with alcohol use, including hypertension, addiction, suicide, accident, and pregnancy complications.

Alcohol is a known carcinogen that may raise cancer risk by acting as a solvent (allowing carcinogens to penetrate the mucosa), an irritant (resulting in increased cell turnover), or possibly a transporter (carrying carcinogens to the basal layer of the mucosa).

It is recommended that people be made aware of the risks and benefits of alcohol use. Individuals who do not drink should not be encouraged to start, and those who do drink should limit alcohol intake to a moderate amount (an average of less than one drink per day for women or less than two drinks per day for men).

## SAFER SEX AND CONTROL OF ONCOGENIC VIRUSES

Unsafe sex is responsible for approximately 2.9 million deaths each year (Ezzati *et al*, 2002) primarily due to the transmission of HIV, but unsafe sex also facilitates the transmission of several other oncogenic viruses (Morrison *et al*, 1997). Human papillomavirus (HPV) causes cervical, vulvar, penile, and anal cancer; hepatitis B and C viruses cause hepatocellular carcinoma; human lymphotropic virus-type 1 is associated with adult T-cell leukaemia; human immunodeficiency virus-type 1 causes Kaposi's sarcoma and non-Hodgkin's lymphoma; and human herpes virus causes Kaposi's sarcoma and body cavity lymphoma.

Prevention strategies to combat the spread of sexually transmitted viruses include behavioural and educational interventions to promote safer sex practices (US Institute of Medicine, 1997) and biomedical programs to develop infection prophylaxis and treatment (Koutsky *et al*, 2002). Regulatory and structural changes are also crucial.

Many of these viruses can also be spread through contact with infected blood, and additional strategies to prevent transmission include needle exchange programmes for intravenous drug users, regulation of tattooing and acupuncture, screening of blood donors, and the development of artificial blood products. In addition, advances in vaccines may offer new avenues for prevention.

## SCREENING

While this review focuses mainly on lifestyle factors that impact the risk of multiple cancers and other chronic diseases, there are additional prevention methods, such as screening, that focus specifically on individual cancers. Screening for cervical and colorectal cancer reduces cancer incidence through the detection and treatment of premalignant conditions. In cases where malignancy has already developed, these tests can decrease mortality by finding cancer at its earliest and most treatable stages. Similarly, by facilitating early detection and treatment, screening for breast cancer (and, in some cases, prostate cancer) can also reduce cancer mortality.

Widespread implementation of screening programmes could significantly reduce the burden of cancer. Issues of access to testing and appropriate follow-up are important at local and national levels.

## SUN PROTECTION

Sun protection is a behavioral factor that specifically reduces the risk of skin cancers and certain types of lip cancer. Sun exposure increases the risk of basal cell carcinoma, squamous cell carcinoma, and malignant melanoma. The incidence of melanoma is rising more rapidly than the incidence of any cancer in the US. Targeting only the subset of the population with high-risk characteristics, such as fair skin or family history, is inadequate for prevention because it fails to identify an adequate proportion of people who develop disease (English and Armstrong, 1988). Instead, it is recommended that all individuals take steps to protect themselves from solar radiation by limiting the time spent under the sun, wearing hats and other protective clothing, and using sunscreen.

Multilevel interventions are required to change social norms to reduce sun exposure in the population. The successful prevention initiative in Australia combined efforts of communities, industry, and government to create educational programmes, media efforts, new products, and policy changes.

## MEDICATIONS

A number of commonly used medications influence the risk of specific cancers. For example, postmenopausal hormone therapy increases the risk of breast cancer, and unopposed oestrogen use raises the risk of endometrial cancer. However, other medications offer cancer protection benefits. Long-term use of oral contraceptive pills decreases the incidence of ovarian and uterine cancers, and regular use of aspirin and other nonsteroidal anti-inflammatory drugs reduces the risk of adenomatous polyps and colorectal cancer. Although not appropriate for all individuals, these medications offer risk reduction benefits for those who take them.

## CONCLUSION

The burden of cancer could be significantly reduced through lifestyle modification.(Table 1

**Table 1:** Key cancer prevention messages

	**Cancer risk reduction benefit**												
**Prevention strategy**	**Bladder**	**Breast**	**Cervical**	**Colorectal**	**Oesophageal**	**Kidney**	**Lung**	**Oral**	**Pancreatic**	**Prostate**	**Skin**	**Stomach**	**Uterus**
*Avoid tobacco use*, including cigarettes, cigars, pipes, and smokeless tobacco products, as well as environmental tobacco smoke.	x		x	x	x	x	x	x	x			x	
*Be physically active*. Get at least 30 min of moderate physical activity on most days of the week.		x		x									
*Maintain a healthy weight*. Balance caloric intake from the diet with energy expenditure from physical activity.		x		x	x	x							x
*Eat a healthy diet* that is rich in fruits and vegetables, is limited in red meat and animal fat, and includes a daily multivitamin with folate.	x	x		x	x		x	x	x	x		x	
*Limit alcohol* to less than one drink per day for women or less than two drinks per day for men.		x		x	x			x					
*Limit your number of sexual partners, and always use condoms*.			x										
*Get appropriate screening tests*.		x	x	x						x			
*Avoid excess sun exposure*. Stay out of the sun as much as possible, wear hats and other protective clothing, and use sunscreen.								x			x		

). Widespread behaviour change would bring tremendous population benefit in terms of reducing the incidence of not only cancer but also other common chronic diseases, such as cardiovascular disease and type II diabetes. However, to realise this large potential benefit, individual healthy choices must be facilitated and reinforced by interventions on multiple levels, including regulatory efforts and environmental changes. It is no longer enough simply to identify behavioural risk factors or set goals for risk reduction, and education alone is not sufficient to motivate behaviour change. It is time to dedicate greater efforts and resources to the implementation of our existing knowledge in order to bring about population-wide behaviour change and significant public health improvements.

For additional information on cancer prevention strategies for individuals and communities, see the Harvard Center for Cancer Prevention website at www.yourcancerrisk.harvard.edu.
